# Development of entrustable professional activities for paediatric intensive care fellows: A national modified Delphi study

**DOI:** 10.1371/journal.pone.0248565

**Published:** 2021-03-18

**Authors:** Marije P. Hennus, Anneliese Nusmeier, Gwen G. M. van Heesch, Maaike A. Riedijk, Nikki J. Schoenmaker, Marijn Soeteman, Enno D. Wildschut, Tim Fawns, Olle Ten Cate

**Affiliations:** 1 Pediatric Intensive Care, University Medical Center Utrecht, Utrecht, The Netherlands; 2 Pediatric Intensive Care, Radboud University Medical Center, Nijmegen, The Netherlands; 3 Pediatric Intensive Care, Erasmus University Medical Center, Rotterdam, The Netherlands; 4 Pediatric Intensive Care, Amsterdam UMC, University of Amsterdam, Amsterdam, The Netherlands; 5 Medical Education Department, Edinburgh Medical School, The University of Edinburgh, Edinburgh, The United Kingdom; 6 Center for Research and Development of Education, University Medical Center Utrecht, Utrecht, the Netherlands; Lundquist Institute at Harbor-UCLA Medical Center, UNITED STATES

## Abstract

Entrustable professional activities (EPAs), as a focus of learner assessment, are supported by validity evidence. An EPA is a unit of professional practice requiring proficiency in multiple competencies simultaneously, that can be entrusted to a sufficiently competent learner. Taken collectively, a set of EPAs define and inform the curriculum of a specialty training. The goal of this study was to develop a set of EPAs for Dutch PICU fellows. A multistage methodology was employed incorporating sequential input from task force members, a medical education expert, PICU fellowship program directors, and PICU physicians and fellows via a modified three-round Delphi study. In the first modified Delphi round, experts rated indispensability and clarity of preliminary EPAs. In the subsequent rounds, aggregated scores for each EPA and group comments were provided. In round two, respondents rated indispensability and clarity of revised EPAs. Round three was used to gain explicit confirmation of suitability to implement these EPAs. Based on median ratings and content validity index (CVI) analysis for indispensability in the first two rounds, all nine preliminary EPAs covered activities that were deemed essential to the clinical practice of PICU physicians. Based on median ratings and CVI analysis for clarity however, four EPAs needed revision. With an agreement percentage of 93–100% for all individual EPAs as well as the set as a whole, a high degree of consensus among experts was reached in the third round. The resulting nine PICU EPAs provide a succinct overview of the core tasks of Dutch PICU physicians. These EPAs were created as an essential first step towards developing an assessment system for PICU fellows, grounded in core professional activities. The robust methodology used, may have broad applicability for other (sub)specialty training programs aiming to develop specialty specific EPAs.

## Introduction

To become a certified Paediatric Intensive Care Unit (PICU) physician, paediatricians or anaesthesiologists in training at any Dutch PICU merely have to provide a ‘satisfactory level of care’ during their 2.5-year fellowship. No official (and/or uniform) forms of assessment are currently in use. However, as this ‘satisfactory level of care’ is not predefined and there are no guidelines on how to achieve this, it is unclear how supervising consultants can perform a valid and reliable assessment of a fellow’s performance. Hence, a recently installed national task force has been asked to develop and implement an official assessment system for Dutch PICU fellows.

As the main goal was to improve a fellow’s performance by providing structured feedback based on the observed performance, a Workplace-Based Assessment (WBA) focusing on assessment for learning [1], was the preferred format. WBAs are a vital component of competency-based medical education (CBME) [1]. CBME, by definition, aims to generate clinicians who meet recognised standards of competence [2]. Medical competence or competency can be defined as a “learnable, durable, and measurable ability to execute a specific, integrative task that is a part of the full range of tasks that constitute the medical profession” [3]. Competencies however, being behavioural descriptors, need a strong link to clinical practice to allow teachers to both observe and use them in assessing a learner’s performance. Entrustable professional activities (EPAs), first introduced in 2005 as a focus of learner assessment, may serve as this much needed link to help bridge the gap between competency frameworks and the challenges of education, assessment and clinical practice [4].

An EPA is a unit of professional practice, or profession-specific task, to be entrusted to a learner (in this case a PICU fellow), once he or she has demonstrated the integration of the required competencies, essential knowledge, and appropriate skills and attitudes [5,6]. Becoming competent in performing an EPA reflects a learner’s journey along five so-called entrustment-supervision scale steps. Each behaviourally anchored, ordinal scale value reflects a judgment that has clinical meaning for assessors [7,8]. This clinical meaning becomes apparent by linking entrustment to levels of supervision. A five-level rating scale for supervision is being used: 1) learners may be present but only observe; 2) learners act under direct supervision; 3) learners act with indirect supervision; 4) learners act unsupervised; and 5) trainees provide supervision to others [9]. This scale has been widely used in Dutch (sub)specialty training programs, including paediatrics, and therefore familiar to both PICU fellows and physicians. Initially, a learner will only be allowed to observe a professional task being performed by a supervisor but over time, as a learner increases in his/her competence and skills, the learner will eventually be trusted to perform the same task unsupervised [6].

Taken collectively, a set of EPAs can be used to define the speciality certificate and form the framework of a curriculum of a specialty training. Such an EPA-based curriculum has the potential to link clinical training and assessment to the work that clinicians actually do in daily practice [10]. Subsequently, an increasing number of postgraduate medical training programs have begun using them to redesign their curricula [11–13].

Five key steps in designing an EPA-based curriculum have been described: 1) identifying suitable EPAs, 2) creating descriptions of the EPAs including a full description of each task and definition of supervision levels, 3) determining assessment frameworks and rules, 4) establishment of individualized pathways with portfolios and 5) allowing for flexibility in length or breadth of training [6]. The essential first steps in developing an EPA-based assessment framework for Dutch PICU fellows requires identifying activities essential to clinical competence of Dutch paediatric intensive care physicians and converting these into an elaborate set of EPAs [14,15]. Consequently, the goal of this study is to identify, develop, and validate the content of a set of EPAs for Dutch PICU fellows using a modified Delphi approach.

## Materials and methods

### Creation of the preliminary draft of EPAs

First, a list of preliminary PICU EPAs was drafted by the national PICU educational task force (five PICU physicians) based on a review of extant EPA literature, the Dutch national training guidelines for PICU fellows, Dutch adult ICU EPAs [16] and the task force members’ own opinions of what constituted essential professional activities of PICU physicians. An iterative process of telephone conferences, emails and a single face-to-face meeting was used to reach consensus on a workable number of EPAs. Hereafter, these EPAs were reviewed by an educational expert for meeting defined EPA criteria [6,17], creating the second draft. Finally, a focus group of all Dutch PICU fellowship training directors refined and finalised the preliminary EPAs in a face-to-face meeting. The resulting third draft of EPAs was used for the content validation process via a 3-round modified Delphi technique as described below.

### Content validation of EPAs using a 3-round modified Delphi technique

As stated by Downing [18], all assessments in medical education, including EPAs as a focus of learner assessment, require evidence of validity to be interpreted meaningfully. Historically, validity referred to the evidence presented to either support or refute the meaning or interpretation of assessment results [18]. Recently however, Taylor et al. [19] recommended that arguments for validity should not necessarily be so restrictive in application. They argue, that in the context of EPAs, evidence of construct validity should include whether developed EPAs correctly and effectively describe the essential work required for clinical practice. Without this tight alignment between developed EPAs and actual professional practice, EPA-based assessments may not accurately capture practice readiness as intended. Subsequently, development and implementation of a set of EPAs must be supported by evidence that defends them as valid descriptions of the profession [20]. In EPA development, content validity arguments focus on the experts involved, the relevance of their expertise and collective representativeness amongst experts for the specialty or profession [19].

A recent systematic review of seven years of research on EPAs in graduate medical education [13] showed that the Delphi technique, allowing for content validation of preliminary EPAs, was part of a favoured multi-step approach to EPA development. The Delphi technique, named with reference to the Ancient Greek god Apollo whose Delphi oracle was viewed as his most expert, truthful and trustworthy informant [21], is a method which establishes expert consensus on a topic, deploying iterative consultation of experts without interaction, allowing for equal weighting of all individual opinions [22,23]. A traditional Delphi typically begins with an open-ended questionnaire that is given to a panel of selected experts. In case of a modified Delphi technique however, the process starts with a set of carefully selected items drawn from various sources e.g., interviews with experts and/or literature reviews [24,25]. This is thought not only to improve the first-round response rate but also provide solid grounding in previously developed work [24,25]. For this particular project, a modified Delphi technique was used as the starting point of the first round consisted of set of carefully drafted EPAs. In addition, the selected expert panel consisted of both novices (PICU fellows) and experts in the field (paediatric intensive care physicians and PICU fellowship training directors).

The purpose and structure of EPAs as well as aims and methods of the modified Delphi study were presented at a quarterly national meeting of Dutch PICU physicians. This was followed by an open discussion to clarify concerns and questions about the project, EPAs, and/or questionnaire. Hereafter, an email containing information on purpose and structure of EPAs, aims and methods of the study and an invitation to participate as a Delphi panelist was send to all practicing PICU physicians (the experts daily involved in training and assessing PICU fellows). By allowing these future users to participate in the development process of this assessment, the resulting EPAs not only reflect a broad practice pattern across clinical settings and regions, but also create a support base for use of the EPAs. Lastly, to ensure input from learners, all PICU fellows were invited to participate in the online questionnaire as well. These fellows are all familiar with EPAs as the current national Dutch paediatric training program, a prerequisite for the fellowship program, is already EPA-based.

The Delphi questionnaire for round one was developed by the national PICU task force based on the final draft of preliminary EPAs and previously published surveys used for EPA development and content validation [20,26,27]. A preliminary draft of this questionnaire was first tested, by way of cognitive interviewing [28,29], on three first year PICU fellows and an adult ICU physician not included in the modified Delphi study. During cognitive interviewing, additional verbal information about responses to draft survey questionnaires was collected from targeted individuals. This information helped evaluate not only the quality of the response but also assist in determining whether these draft questions are indeed providing (or are failing to provide) the desired information [29]. Based on cognitive interviewing of the four individuals mentioned above, only some minor textual revisions were deemed necessary.

The resulting first-round Delphi questionnaire started with a general introduction of the study, followed by an extensive information letter and an electronic informed consent form (both in Dutch). Electronic written informed consent was mandatory to allow for progression to the actual study which started with a (Dutch) podcast (https://www.youtube.com/watch?v=ZIyGBcomc7Q&t=2s). The content of this podcast was similar to the content of the presentation given during the quarterly national meeting of Dutch PICU physicians, hereby potentially ensuring a basic level of knowledge across all panellists. Hereafter, demographic data for each panellist were collected. In addition, preliminary EPAs (title, description, and detailed specifications) were listed and panellists were asked to rate to what extent each EPA reflected an essential task for a PICU physician (‘indispensability") on a five-point Likert-scale [30]. ‘Clarity’ of the description of each EPA was to be rated on a second 5-point Likert scale and an open text box for each EPA allowed for feedback and elaboration of provided answers. Finally, panellists were asked if the list of EPAs fully described the specialty of a Dutch PICU physician (‘comprehensiveness’; five-point Likert-scale) and/or to elaborate on any additional EPAs that should be added (open text box). After round one, two task force members reviewed and categorised comments, resolved possible differences by consensus and if necessary revised EPAs.

In round two, titles and descriptions of revised EPAs were sent to the responding panellists from round one together with results of the first round. Panellists were asked to re-rate revised EPAs for indispensability and clarity using the same scales as in round one. A single comment box solicited written feedback on each EPA. In addition, panellists were asked if the list of EPAs, now containing the revised EPAs, was comprehensive (five-point Likert scale) and to identify any EPAs that should be added (open text box). Comments were reviewed and resolved in a process identical to round one.

In round three, results from round two were used as feedback to panellists and agreement with the near-final list of PICU EPAs as method of fellow assessment was sought for each individual EPA and for the complete list of EPAs.

Castor (Electronic Data Capture)^®^ software was used to construct and automate the modified Delphi questionnaires.

#### Data analysis

In the first two rounds, median and content validity index (CVI) for ‘indispensability’ and ‘clarity’ were calculated for the Likert scale data of each EPA as well as the median and CVI for the Likert scale data of ‘comprehensiveness’ of the set as a whole. The CVI, the degree to which an instrument has an appropriate sample of items for the construct being measured [31], was computed as the number of panellists giving one of the highest two ratings for each EPA, divided by the total number of panellists. CVI values can range from 0 to 1: as a cut-off score, we determined that a CVI of 0.8 or higher indicated sufficient content validity, a CVI within the range 0.70 and 0.79 implied that the item required revision, and a CVI below 0.70 indicated elimination of the corresponding EPA [32,33]. If the median for an EPA were below the predetermined consensus level of 4 (out of the 5-point Likert scale with level 4 and 5 corresponding with two highest agreement levels) for either ‘indispensability’ and/or ‘clarity’, revision of that particular EPA would be required. For round three, a predetermined consensus percentage for each individual EPA and the set as a whole was set to 80% or higher. All data were analysed using R statistical software, version 3.6.2 (2019-12-12) [34].

### Ethical approval

The Ethical Review Board of the Netherlands Association of Medical Education approved this study (ERB number 2019.3.5). Prior to participation, all participants received an online information letter and were asked for written consent. All collected data was pseudonymised, saved electronically on encrypted storage devices in a secure location and destroyed once no longer in use.

## Results

### Developing preliminary EPAs

Nine preliminary EPAs were developed by the national PICU task force. The first five involved assessing and treating patients of differing complexity and acuteness within the PICU. The remaining four EPAs covered additional essential activities (e.g., multidisciplinary communication, managing complex situations). Besides a title, each EPA consisted of a detailed description, required knowledge, a link with the prevailing CanMEDs framework of competencies [35], skills and attitudes, methods of assessing progress, suggested entrustment conditions, and criteria for arriving at an entrustment decision as described by ten Cate [36]. Hereafter, EPAs were reviewed by an educational expert and refined and finalised by a focus group consisting of all Dutch PICU fellowship training directors (n = 7). No modifications in the number or breadth of EPAs were considered necessary and only minor textual alterations were made. The titles and detailed descriptions of the resulting third draft of EPAs were used in round one of the ensuing modified Delphi study.

**Fig 1:**
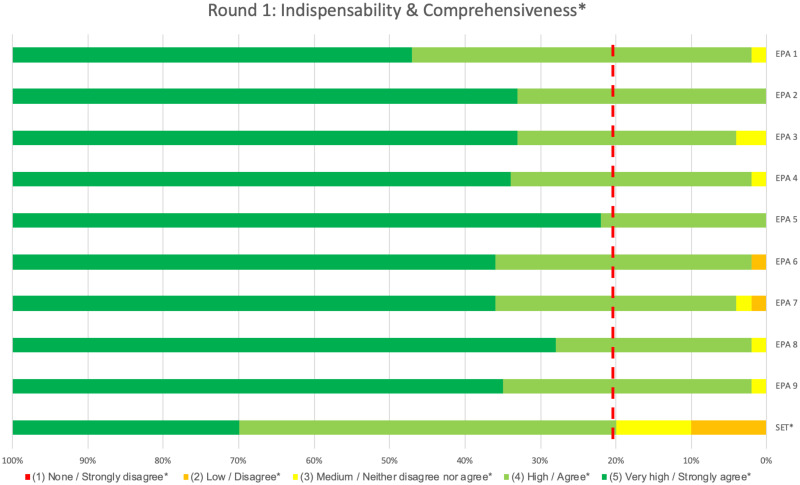
Original draft of EPA 6. Initial draft of EPA 6 (translated into English) as presented to panellists in the 1^st^ modified Delphi round.

To illustrate the developmental and validation process as well as to help overcome the fact that all actual EPA descriptions and responses from the panellists are in Dutch, one EPA, namely EPA 6 ‘Assessing and treating an acutely ill patient outside of the PICU’ is translated into English and followed through each modified Delphi round to show how it evolved. Fig 1 shows the initial draft of EPA 6 as presented to panellists in the first Delphi round.

### Modified Delphi study

#### Round one

In round one, 58 (63%) of the 92 questionnaires that were sent out to all Dutch PICU physicians (n = 70) and fellows (n = 22), were fully completed by the respondents (Table 1). Four questionnaires were only partially completed and therefore not included in the data analysis of this first round. The typical profile of the participants was between 40–50 years of age (41%), most females (64%), paediatricians (86%), employed as staff members (90%) with less than five years of experience working on a PICU (28%) (Table 1).

**Table 1:** Demographics of respondents of the three Delphi rounds.

Responders			
	Round 1	Round 2	Round 3
**Questionnaires, n**			
Total sent/fully completed (%)	92/58 (63%)	62/51 (82%)	62/46 (74%)
Fellows sent/fully completed (%)	20/6 (30%)	6/5 (83%)	6/5 (83%)
Staff sent/fully completed (%)	72/52 (72%)	52/46 (89%)	52/41 (79%)
**Age in years, n (%)**			
< 30	0 (0%)	0 (0%)	0 (0%)
30–40	17 (30%)	15 (29%)	14 (30%)
40–50	24 (41%)	24 (47%)	19 (41%)
50–60	13 (22%)	9 (18%)	10 (22%)
> 60	4 (7%)	3 (6%)	3 (7%)
**Sexe**			
Female	37 (64%)	35 (69%)	31 (68%)
Male	20 (34%)	16 (31%)	14 (30%)
Other	1 (2%)		1 (2%)
**Specialty, n (%)**			
Paediatrics	50 (86%)	43 (84%)	38 (83%)
Anaesthesiology	8 (14%)	8 (16%)	8 (17%)
**Position**			
Fellow	6 (10%)	5 (10%)	5 (11%)
Staff	52 (90%)	46 (90%)	41 (89%)
**Years of Experience**			
< 5	16 (28%)	15 (29%)	13 (28%)
5–10	11 (19%)	10 (20%)	9 (20%)
10–15	11 (19%)	11 (22%)	8 (17%)
15–20	8 (14%)	7 (14%)	6 (13%)
> 20	12 (20%)	8 (15%)	10 (22%)

**Fig 2:**
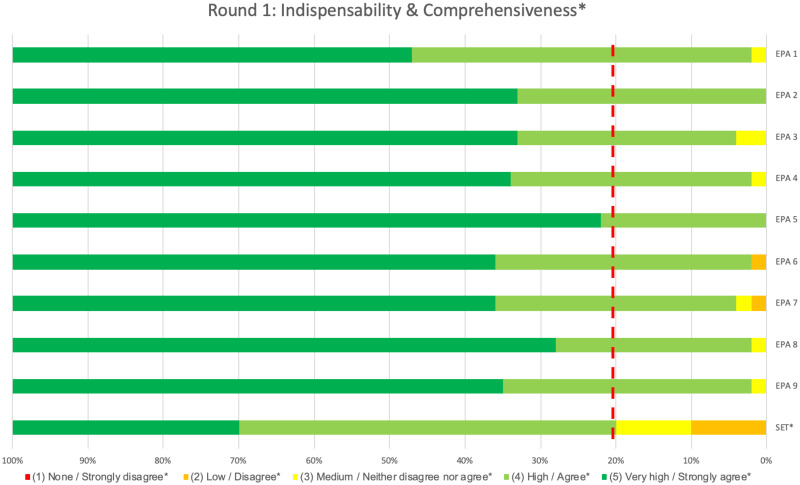
Indispensability and comprehensiveness results of the 1^st^ Delphi round. The proportion of panellists rating each of the 5 Likert scale points for ‘Indispensability (‘none’, ‘low’, ‘medium’, ‘high’, ‘very high’) of each individual EPA and ‘Comprehensiveness’ (‘strongly disagree’, ‘disagree’,’ neither disagree nor agree’, ‘agree’, ‘strongly agree’) of the set as a whole in round 1 are shown. The dotted red line shows the threshold value of 80%, all ratings to the left of this line should either be ‘high/agree’ (light green) or ‘very high/strongly agree’ (dark green) to indicate sufficient content validity.

In all rounds, no significant difference in median ratings and/or content validity index (CVI) were found between fellows and staff members, therefore results are reported for the two groups combined. In the first round, ‘indispensability’ results for all individual EPAs met predefined thresholds. Not only did all medians equal the maximum rating of five, each of the nine EPAs also had a calculated content validity index (CVI) of 0.8 or higher (Table 2). This demonstrated that, according to the respondents, all proposed EPAs indeed covered activities essential to clinical practice of Dutch PICU physicians and no essential PICU activity was missing from the proposed set. These findings were further accentuated by the percentage of respondents rating the higher end, namely ‘high’ and ‘very high’, of the five different scale points for ‘indispensability’ (Fig 2). Subsequently, no revisions were deemed necessary based on the ‘indispensability’ data alone.

**Table 2:** Indispensability, comprehensiveness and clarity results of the 1^st^ two Delphi rounds and agreement data of the 3^rd^ round. Median and Content Validity Index (CVI) for ‘Indispensability’ and ‘Clarity’ of individual EPAs as well as ‘Comprehensiveness’ (*) for the set as a whole of the first two Delphi rounds. Delphi round three agreement of panellists with the proposed (set of) EPAs in percentage and absolute numbers. Values below the predetermined thresholds are marked orange.

	Median Indispensability (Comprehensiveness set*)		CVI Indispensability (Comprehensiveness set*)		Median Clarity		CVI Clarity		Percentage (absolute number)	
EPA number	Round 1	Round 2	Round 1	Round 2	Round 1	Round 2	Round 1	Round 2	Agree	Disagree
EPA 1	5		0,98		4		0,86		100 (46)	0 (0)
EPA 2	5		1,00		4		0,83		100 (46)	0 (0)
EPA 3	5		0,96		4		0,84		100 (46)	0 (0)
EPA 4	5	5	0,98	0,96	4	4	0,78	0,90	100 (46)	0 (0)
EPA 5	5		1,00		5		0,85		100 (46)	0 (0)
EPA 6	5	5	0,98	0,98	4	4	0,74	0,88	96 (44)	4 (2)
EPA 7	5	5	0,96	0,98	4	4	0,79	0,94	100 (46)	0 (0)
EPA 8	5		0,98		4		0,83		93 (43)	7 (3)
EPA 9	5	5	0,98	0,96	4	4	0,79	0,92	100 (46)	0 (0)
Set*	4	4	0,79	0,98					96 (44)	4 (2)

**Fig 3:**
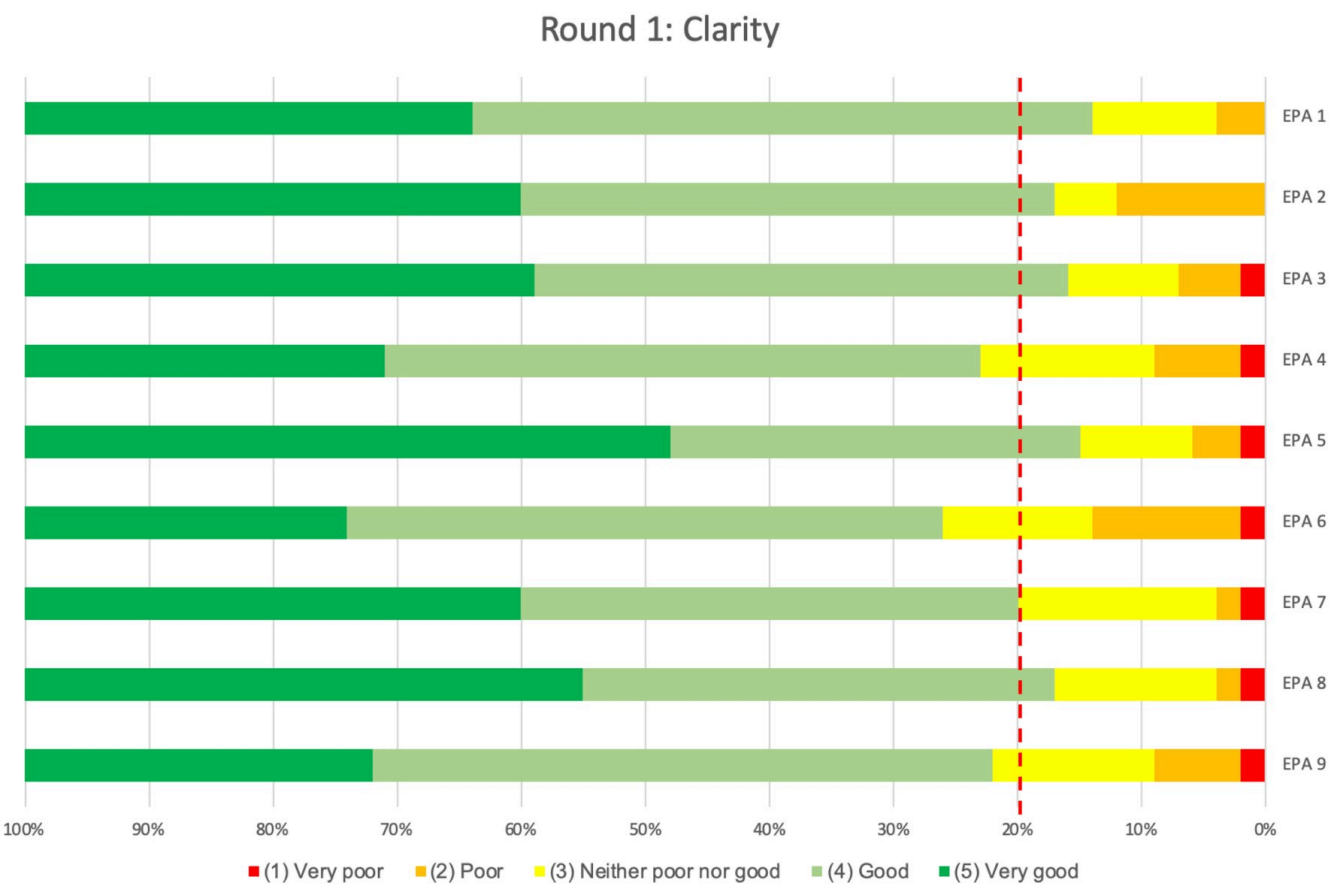
Clarity results of the 1^st^ Delphi round. The proportion of panellists rating each of the 5 Likert scale points for ‘Clarity’ (‘none’, ‘low’, ‘medium’, ‘high’, ‘very high’) for each individual EPA in round 1 are shown. The dotted red line shows the threshold value of 80%, all ratings to the left of this line should either be ‘good’ (light green) or ‘very good’ (dark green) to indicate sufficient content validity.

In contrast to the ‘indispensability’ results, respondents found descriptions of four out of these nine EPAs lacked ‘clarity’. Despite all medians meeting the predefined threshold, this lack of ‘clarity’ was demonstrated by an insufficient CVI, below the threshold of 0.8, for EPA 4 (0.78), EPA 6 (0.74), EPA 7 (0.79) and EPA 9 (0.78) (Table 2). Furthermore, a higher percentage of respondents rated the middle and lower end (‘neither poor nor good’, ‘poor’ or ‘very poor’) of the five different scale points for ‘clarity’ for these specific EPAs (Fig 3).

Although the set’s median for ‘comprehensiveness’ was appropriate, its CVI did not meet the predefined threshold of 0.8 (Table 2). Insufficient content validity was therefore assumed for the set as a whole. This was also reflected in the proportion of panellists rating the middle and lower scale points of ‘comprehensiveness’ (Fig 2). Despite not meeting the threshold CVI for ‘comprehensiveness’, the set was not revised prior to round two, as revision of the four individual EPAs on the basis of their clarity indices was expected to (positively) influence the set’s ‘comprehensiveness’ rating.

**Fig 4:**
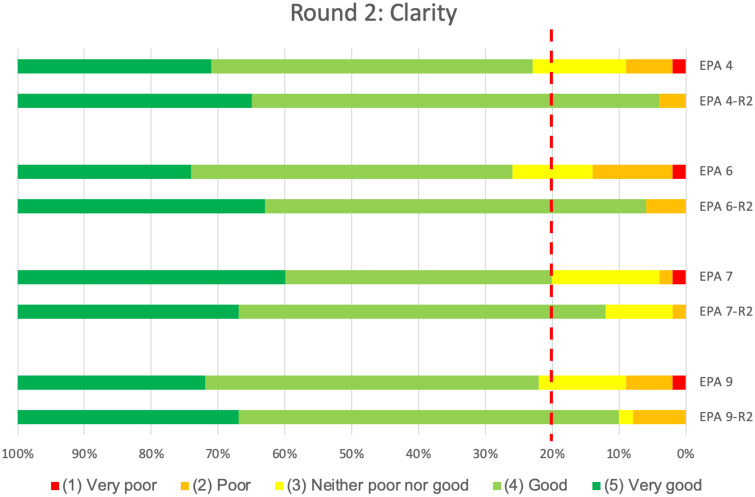
The revised EPA 6. The revised EPA 6 (translated into English) as used in the 2nd Delphi round with track changes in red.

Given these aforementioned ‘clarity’ results, EPA’s 4, 6, 7 and 9 were revised prior to the second Delphi round based on the panellists’ suggestions. Two task force members (MH and AN), reviewed and categorised every written comment, determined eligible alterations, and resolved possible differences by consensus. Comments were allocated to one of the following categories with ensuing consequences: a) suggestion regarding textual clarifications and/or alterations: suggestion accepted if unanimously agreed on by the two task force members; b) suggestion contradicting extant EPA guidelines [6]: suggestion rejected; c) suggestion regarding content of an EPA made by ≥ 5% of all panellists: suggestion accepted and d) suggestion regarding content of an EPA made by < 5% of all panellists: suggestion rejected. In addition to these EPA-specific comments, more general comments revolved around emphasizing the differences among the six patient-related EPAs. These inter-EPA differences were emphasised by revising the ‘detailed descriptions and limitations section’ to include specifics of what a fellow should know and or do for a particular EPA, and making the ‘specific knowledge, skills and attitude’ section into a more general description (Fig 4). Hereafter, the four revised EPAs, with track changes in red, a written summary of why each change was made as well as a copy of all the original comments from the panellists in this first round were sent out for approval to the national PICU task force. After their unanimous approval, these four revised EPAs, with bold and/or red text clearly showing all revisions, were used in the second Delphi round.

#### Round two

**Fig 5:**
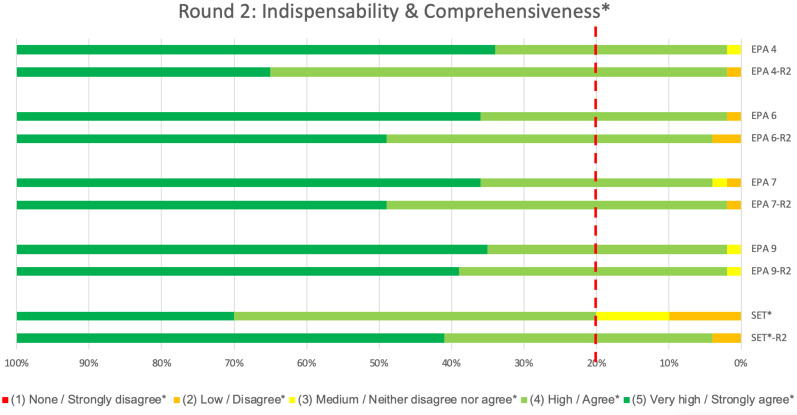
Comparison of indispensability and comprehensiveness results of the 1^st^ and 2^nd^ Delphi round (revised EPAs only). The proportion of panellists who rated each of the 5 Likert scale points for ‘Indispensability’ (‘none’, ‘low’, ‘medium’, ‘high’, ‘very high’) for the four revised EPAs as well as ‘Comprehensiveness*’ (‘strongly disagree’, ‘disagree’,’ neither disagree nor agree’, ‘agree’, ‘strongly agree’) of the set as a whole in round 2 (-R2) are shown compared to their results of the 1^st^ round. The dotted red line shows the threshold value of 80%, all ratings to the left of this line should either be ‘high/agree’ (light green) or ‘very high/strongly agree’ (dark green) to indicate sufficient content validity.

The second-round questionnaire, as well as the set of EPAs (including the four revised ones), was sent out to the respondents of the first round. In this second round, 51 (82%) of the 62 questionnaires sent, were completed by the respondents (Table 1). There were no partially completed questionnaires. The most common characteristics of respondents in round two were similar to round 1 (Table 1). Revision of the four EPAs did not impact their score for ‘indispensability’ as, similar to round one, the round two medians and CVIs for ‘indispensability’ still met the predefined thresholds. However, the revised EPAs positively influenced the set’s second round ‘comprehensiveness’ results, as both the set’s median and CVI were now above predefined thresholds of 0.8 as well (Table 2). This was also reflected by a higher proportion of respondents assigning higher scale points for ‘indispensability’ of individual EPAs and ‘comprehensiveness’ of the set (Fig 5).

**Fig 6:**
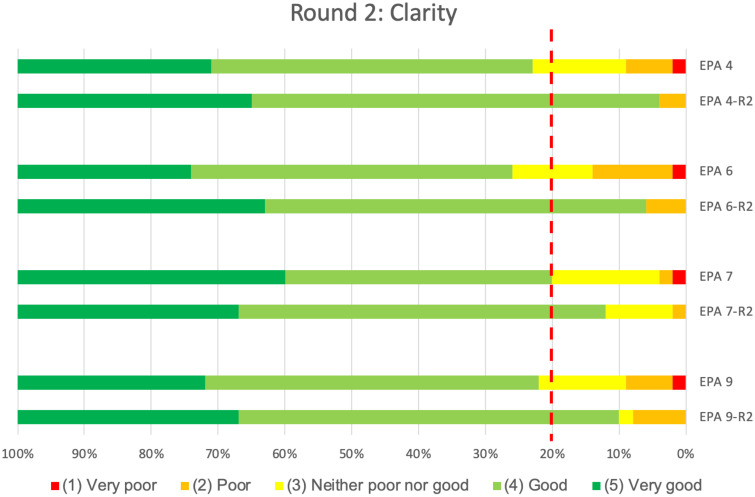
Comparison of clarity results of the 1^st^ and 2^nd^ Delphi round (revised EPAs only). The proportion of panellists rating each of the 5 Likert scale points for ‘Clarity’ (‘none’, ‘low’, ‘medium’, ‘high’ or ‘very high’) for the four revised EPAs in round 2 (-R2) are shown compared to their results of the 1^st^ round. The dotted red line shows the threshold value of 80%, all ratings to the left of this line should either be ‘good’ (light green) or ‘very good’ (dark green) to indicate sufficient content validity.

Notably, revision of these four EPAs also positively impacted the clarity of their descriptions. In contrast to the first-round results, in this second round both median and CVI for all four revised EPAs were above the predefined thresholds of 0.8, indicating sufficient content validity for ‘clarity’ (Table 2). Furthermore, a higher proportion of respondents assigned the higher scale points for ‘clarity’ of individual EPAs (Fig 6).

Based on the positive round-two results for ‘indispensability’, ‘clarity’ and ‘comprehensiveness’, no additional revisions of either individual EPAs or the set were deemed necessary. Nevertheless, the third round was still needed given its political aspect of gaining explicit confirmation to implement the proposed (set of) EPAs.

#### Round three

In this last round, 46 (74%) of the 62 questionnaires that were sent out were fully completed by the respondents and demographics of respondents were in line with the first two rounds (Table 1). Notably, an overwhelming majority agreed with implementation of all individual EPAs and the set as a whole as a focus of fellow assessment with the lowest percentage of agreement, 93% for EPA 8, still being amply above the predefined acceptance rate of 80% (Table 2). This resulted in the following set of nine PICU EPAs:

1. Assessing and treating a not acutely ill, stable, low complexity patient
2. Assessing and treating a not acutely ill, stable, high complexity patient
3. Assessing and treating an acute problem of a previously stable patient
4. Assessing and treating a high complexity patient with a relatively simple and treatable acute problem
5. Assessing, treating an acutely ill, unstable, high complexity patient
6. Assessing and treating and/or transporting an acutely ill patient outside of the PICU
7. Communicating with patients, parents/caregivers and/or other healthcare professionals
8. Performing skills essential for PICU physicians
9. Managing complex situations (on a PICU)

**Fig 7:**
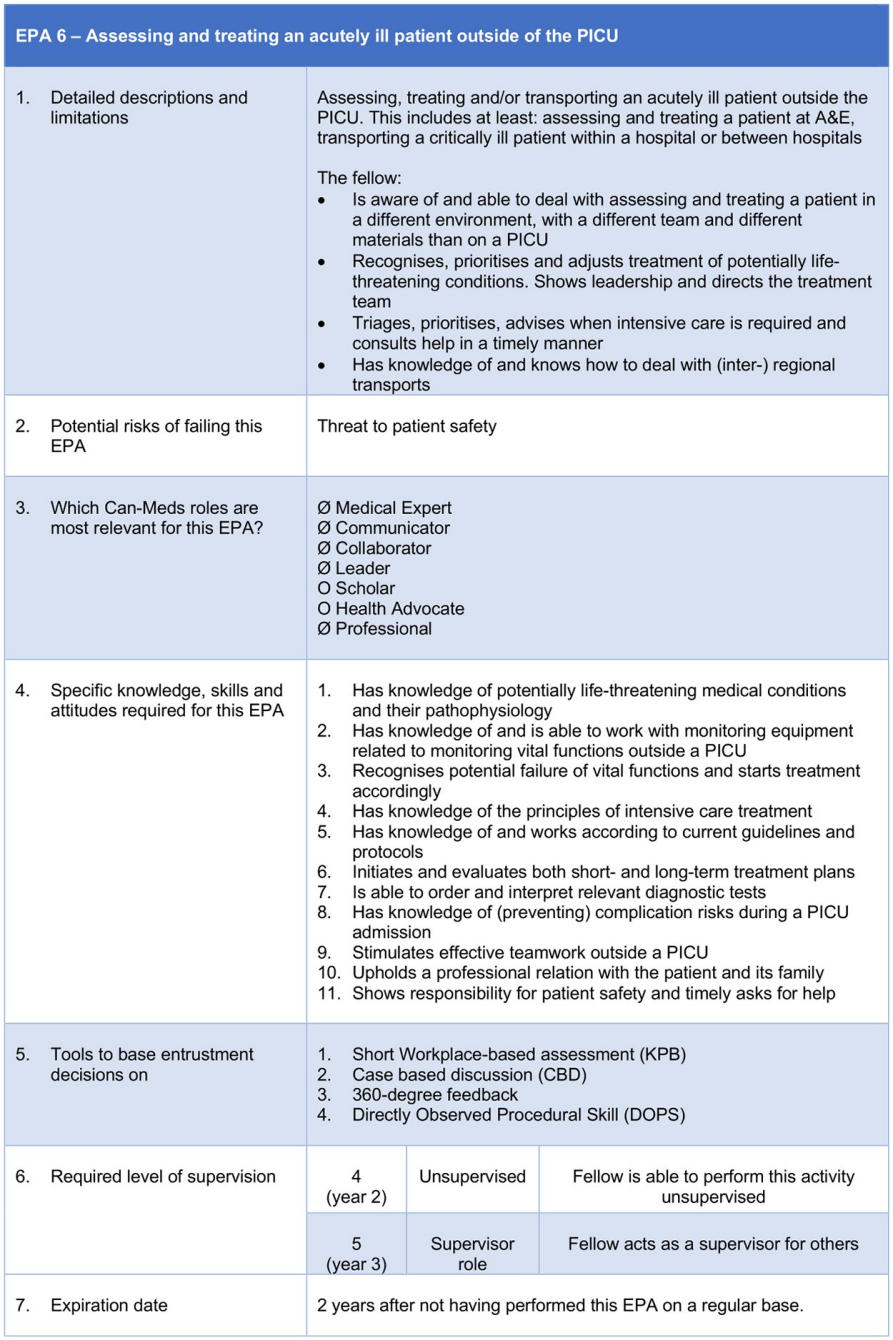
EPA 6: ‘Assessing and treating an acutely ill patient outside of the PICU’. This figure shows the end result of one of the nine final EPAs, namely EPA 6 ‘Assessing and treating an acutely ill patient outside of the PICU’, as it will be used for assessing Dutch PICU fellows.

Besides the above-mentioned titles, each EPA further consisted of a detailed description, required knowledge, skills and attitudes, methods of assessing progress, suggested entrustment conditions, and approach to arriving at an entrustment decision. One of these nine EPAs, which will be used to develop an assessment of Dutch PICU fellows, was translated as an example (Fig 7).

## Discussion

This study describes the identification, development and content validation of a set of EPAs for paediatric intensive care fellows. The multistage methodology employed consensus decision making by a five-member national PICU task force. The results incorporate sequential input from various sources: task force members via iterative deliberation; an expert in medical education via digital consultation; all Dutch PICU fellowship program directors via a focus group; and Dutch PICU physicians via a modified three-round Delphi study. An interesting feature of this study is the involvement of relevant learners, namely PICU fellows, in the modified Delphi process as well. Subsequently, a combined total of 46 experts, including fellows, ultimately reached consensus on a list of nine EPAs thought to comprise a comprehensive range of essential professional daily activities of PICU physicians. These essential activities include among other assessing and treating critically ill patients, performing skills indispensable for PICU physicians and managing complex situations on a PICU. With 96% agreement on the final list of EPAs, a high degree of consensus among experts in paediatric intensive care was reached. The resulting EPAs not only provide a succinct overview of the core tasks of Dutch PICU physicians but also serve as a set of goals and reminders of what PICU fellows must achieve. Furthermore, they will form the basis of an assessment system grounded in core professional activities.

This study resembles previously published studies on developing and validating EPAs for a number of postgraduate medical specialties training programs [20,26,27,37]. Similarities include not only the ‘target group’ of postgraduate trainees but also the range of methods used to develop and validate these EPAs. The methodology for the present study is further substantiated by a recent systematic review of seven years of research on EPAs in graduate medical education [13]. Although the authors of the systematic review were unable to determine an apparent ‘best’ method and considerable variation existed in the developmental process of EPAs, the majority of included papers applied comparable multiple distinct methods. Interestingly, the favoured approach seemed to be a sequential multi-step approach of literature review, drafting preliminary EPAs by a working groups and refining and revising these EPAs via stakeholder deliberation or a (modified) Delphi approach [13], matching the methodology of our study.

The suggested EPAs of the aforementioned studies on postgraduate medical training vary widely in scope and number, ranging from 10 for psychiatry [20] to an astounding 45 for anaesthesiology [27]. With ultimately nine EPAs, the present study clearly is at the lower end of this range. Although ten Cate and Scheele (2007) [38] originally proposed that 50 to 100 EPAs might be needed to cover a specialty’s training program, they lowered that estimate to a maximum of 20 to 30 EPAs for graduate medical education a few years later [39]. A larger number than this recommended maximum is thought to result in very specific and narrow EPAs with the subsequent risk of reinstating issues related with Competency Based Medical Education (e.g., high administrative load, reducing everything to a checklist of competencies) [40,41]. Alternatively, a relatively modest number of broad EPAs that link to multiple competencies and reduce the complexity of the curriculum, as was achieved in the current study, enable a more holistic view of the learner and lend themselves better to implementation and subsequent sustaining of change among all involved [10,42]. Notably, the shorter duration of the PICU subspecialty training program, namely two and a half years, as compared to five-year specialty training programs in the Netherlands (e.g., paediatrics, psychiatry, anaesthesiology), may also have played a role in reaching this modest number of EPAs for PICU fellows. Similarly, the American Board of Pediatrics (ABP) has also implemented a relatively modest number of ten EPAs for their pediatric critical care medicine fellows [https://www.abp.org/subspecialty-epas].

The methodology of the present study included multiple validity-enhancing strategies that might also be relevant to future EPA-related research in any specialty. First, various sources of input were incorporated in the processes of development and validation. These include extant literature on EPAs, PICU task force members, an expert in medical education, all PICU fellowship program directors and PICU physicians. In addition, learners, i.e. PICU fellows, were also explicitly invited to participate in the content validation process. Although this has not been described before, the rationale is twofold. Not only does it prevent drafted EPAs from predominantly reflecting practice patterns of its developers and/or PICU staff, but the active and participatory approach of including learners in the curriculum design also has the potential to enhance and support learning [43]. Unfortunately, only a small percentage (30%) of all fellows responded, comprising 10% of all respondents, but their ratings did not differ from those of staff members. Subsequently, the resulting EPAs were presumed to reflect practice patterns of both learners and supervisors.

Secondly, presumed complexity and acuteness of patients was used as the logic, defined as the perspective used by the developers to break down the practice of a professional domain in units, to subdivide the typically very heterogenous PICU population into five patient categories. These five patient-related EPAs were supplemented with four additional EPAs to cover essential activities of PICU physicians not yet covered. The resultant limited number of nine comprehensive EPAs is thought to facilitate implementation and subsequent sustainment of change among those involved [10,42]. Broad and comprehensive EPAs, linked to multiple competencies, can reduce the complexity of the curriculum and enable a holistic view of the learner [10]. The ten EPAs for American pediatric critical care medicine fellows consist of seven individual EPAs, common to all subspecialties, supplemented with three specific paediatric critical care medicine EPAs. Interestingly, the logic of these seven common EPAs was based on the framework of physician competencies organized around the seven physician roles of the CanMEDS framework [35]. Although these competencies, being personal descriptors such as medical expert, communicator and/or collaborator etc., are extremely important and must be evaluated, our EPAs intend to bring together these multiple competencies into relevant tasks of the profession [4,44].

Finally, by inviting all task force members and program directors as potential panellists in the modified Delphi study, educators with considerable knowledge of the EPA concept also had the opportunity to contribute to the decision-making process. This was in line with a recently developed comprehensive practice analysis incorporating all the skills of a pediatric intensivist throughout the lifespan of a career [45]. This analysis, which started with the extant ABP EPAs for pediatric critical care and expanded on them, sought broad feedback from board-certified pediatric intensivists throughout the United States of America [45].

Along with its strengths, this study has several limitations, many of which are inherent to the Delphi process itself. First, we opted for a modified Delphi approach in which task force members familiar with PICU fellow education and assessment drafted the preliminary EPAs. Their beliefs of what comprises an EPA, even if informed by evidence, gave direction to development of the PICU EPAs. Although this might not necessarily be a weakness, it is important to recognise that different results might have been obtained if a traditional Delphi-approach, starting with an open-ended questionnaire amongst all participants, had been used. That would however have required extensive instruction of Delphi panel members, not all of whom were sufficiently familiar with EPAs. Furthermore, the outcome might have also varied according to which people chose to participate.

Secondly, as with all questionnaire studies, the results of a (modified) Delphi study depend on how the proposition is expressed and only express what is presented. Variation may have remained in understanding what constitutes an EPA in scope and/or activity. This was thought to be reflected by some panellists’ comments on EPA 8 ‘Performing skills essential for PICU physicians’. Of the 30 panelists’ providing comments/suggestions for this EPA, 16 suggested splitting this EPA in multiple smaller EPAs, preferably a separate EPA for each intervention that a PICU physician should be able to perform, e.g., oral/nasal intubation, central venous access, peripheral arterial access, pleural drain placement etc. This demand for multiple, very specific and narrow EPAs that could also be collated in one more general EPA however, risked reducing EPAs to a checklist of competencies with a high administrative load [40,41]. Subsequently, this suggestion was rejected by the task force. Conversely, there is always a risk that suggestions made by panellists were overly marginalised or discounted based on the task force members’ beliefs of what constitutes an EPA, even if these beliefs were theoretically grounded (e.g., by EPA guidelines). According to the respondents however, all proposed EPAs indeed covered activities essential to clinical practice of Dutch PICU physicians and no essential PICU activity was missing from the proposed set. Furthermore, the thorough revision of clarity of descriptions of four of these nine EPAs prior to the second Delphi round, was solely based on the panelists’ suggestions. Finally, an overwhelming majority agreed with implementation of all individual EPAs, including the four revised EPAs, and the set as a whole in the third round. Ultimately, judgement bias remains a limitation of any consensus-based method including the Delphi [46].

Thirdly, while previous studies were followed in deeming a rating ‘high or very high’ or ‘good or very good’ among 80% or more of the participants (corresponding with a CVI above 0.79) as sufficient for retention [32,33], this or any other Delphi criterion for accepting or rejecting a proposition is, unavoidably, arbitrary.

In the end, creating a set of EPAs is a complex process, inevitably subjected to some form of bias, without a clear ‘one size fits all’ solution as reflected by the wide range in number, scope and breadth of EPAs between and within professions, subspecialties and countries [12,13]. All things considered however, the resulting nine EPAs provide an essential and reasonable first step towards an EPA-based assessment program for Dutch PICU fellows, especially as other studies built on a very similar model have proved successful [20,26,27,37].

## Conclusions

This study outlines the development and validation of a set of EPAs for Dutch PICU fellows. First, a list of nine preliminary PICU EPAs was drafted by the national PICU educational task force. This preliminary list of EPAs was then reviewed by an educational expert and refined by a focus group of PICU fellowship directors. In the subsequent three-round Delphi study, Dutch PICU physicians and fellows, reached near uniform agreement on the final list of EPAs. The resulting nine PICU EPAs provide a succinct overview of the core tasks of PICU physicians in the Netherlands and are to form a reasonable first step towards an assessment system for Dutch PICU fellows grounded in core professional activities. The robust methodology used, may have broad applicability for other (sub)specialty training programs aiming to develop and validate specialty specific EPAs.

Future directions include setting performance criteria by which to judge a fellow’s readiness to progress to the next level of entrustment and by establishing which combination of assessment tools would complement the proposed EPAs as part of a holistic assessment programme. Hereafter, implementation, workplace-based testing and evaluation of not only EPAs but also their intended outcome could be useful next steps as part of a plan-do-check-act circle, vital to successful implementation of any project.

## Supporting information

S1 File PICU EPAs in Dutch Plosone. (DOCX)

S2 File Questionnaire Delphi round 1–3. (DOCX)

S3 File Raw data set PICU EPA round 1 –translation. (XLSX)

S4 File Raw dataset PICU EPA round 3 –translation. (XLSX)

S5 File Raw dataset responders PICU EPA round 2- translation. (XLSX)

S6 File Vragenlijst Delphi ronde 1–3. (DOCX)
