# Supplementary material for: Development of entrustable professional activities for paediatric intensive care fellows: A national modified Delphi study
Source: PLoS One. 2021 Mar 18;16(3):e0248565. doi: 10.1371/journal.pone.0248565 (PMC7971696; doi:10.1371/journal.pone.0248565)
Supplement: S1 File — (DOCX) [file pone.0248565.s001.docx]

**PICU EPA’s**

De 9 PICU EPA’s die de kernactiviteiten van een kinderintensivist omschrijven en de rode draad vormen van PET2020 zijn:

1. *Opvang en behandeling van een niet-acuut zieke, stabiele, laag-complexe patiënt*
2. *Opvang en behandeling van een niet-acuut zieke, stabiele, hoog-complexe patiënt*
3. *Opvang en behandeling van een acuut probleem bij een voorheen stabiele patiënt*
4. *Opvang en behandeling van een hoog-complexe patiënt met een relatief eenvoudig te behandelen acuut probleem*
5. *Opvang en behandeling van een acuut zieke, instabiele, hoog-complexe patiënt*
6. *Opvang, behandeling en/of transport van een acuut zieke patiënt buiten de PICU*
7. *Communicatie met kind, ouders en andere betrokken zorgverleners*
8. *Uitvoeren van verrichtingen voor een PICU-arts*
9. *Managen van complexe situaties (op de PICU)*

| EPA 1 – Opvang en behandeling van een niet-acuut zieke, stabiele, laag-complexe patiënt | | | |
| --- | --- | --- | --- |
| 1. Gedetailleerde beschrijving en beperkingen | Opvang en/of behandeling van een **niet-acuut zieke, stabiele, laag-complexe patiënt** op de PICU.  Hiertoe behoren bijvoorbeeld: beademde en niet-beademde postoperatieve patiënt (na electieve ingreep), observatie na milt-/leverlaceratie, chronische patiënt, patiënt met tracheacanule en luchtweginfectie. | | |
| 1. Potentieel risico bij niet goed beheersen van deze EPA | Gevaar voor de kwaliteit van zorg voor de patiënt | | |
| 1. Welke CanMeds competentiegebieden zijn het meest relevant voor deze EPA? | Ø Medisch handelen  Ø Communicatie  Ø Samenwerking  Ο Leiderschap  Ø Kennis en Wetenschap  Ο Maatschappelijk handelen  Ø Professioneel gedrag | | |
| 1. Welke specifieke kennis, vaardigheden en attitudes zijn er nodig om de EPA op het gewenste niveau te beheersen? | 1. Heeft kennis van aandoeningen waarbij de vitale functies potentieel bedreigd kunnen raken, inclusief de achterliggende pathofysiologie 2. Heeft kennis van en vaardigheid met standaard PICU-apparatuur met betrekking tot beademing, bewaking en monitoring van vitale functies en het toepassen hiervan 3. Herkent bedreiging van vitale functies en start behandeling 4. Heeft kennis van de principes van de standaard ic-behandeling 5. Werkt volgens de geldende richtlijnen en protocollen 6. Maakt en (her-)evalueert een behandelplan voor korte en lange termijn 7. Kan diagnostische tests en consulten aanvragen en interpreteren 8. Heeft kennis van de risico’s op complicaties en preventieve maatregelen bij een verblijf op de PICU 9. Stimuleert effectief teamwork op de PICU 10. Onderhoudt een professionele relatie met de patiënt en diens familie 11. Neemt verantwoordelijkheid voor patiëntveiligheid en vraagt op tijd hulp | | |
| 1. Suggesties om voortgang en een ‘entrustment decision’ op te baseren | 1. Korte Praktijk Beoordeling (KPB) 2. Case based discussion (CBD) 3. 360 graden feedback 4. Directly Observed Procedural Skill (DOPS) | | |
| 1. Te behalen supervisie niveau | 4  (6 mnd) | Uitgestelde supervisie | Fellow mag activiteit zelfstandig uitvoeren en rapporteert nadien. Kan geleidelijk overgaan naar ongesuperviseerd uitvoeren van activiteit |
|  | 5  (jaar 1) | Geeft supervisie | Fellow geeft supervisie aan collegae |
| 1. Expiratiedatum | 2 jaar na het niet uitgevoerd hebben van deze EPA | | |

| EPA 2 – Opvang en behandeling van een niet-acuut zieke, stabiele, hoog-complexe patiënt | | | |
| --- | --- | --- | --- |
| 1. Gedetailleerde beschrijving en beperkingen | Opvang en/of behandeling van een **niet-acuut zieke, stabiele, hoog-complexe** **patiënt** op de PICU.  Hiertoe behoren tenminste: stabiele patiënt met complex onderliggend lijden, stabiele patiënt met multi-orgaan problematiek, stabiele pre-/postoperatieve cardio-chirurgische patiënt, stabiele dialyse  **De fellow:**   - Integreert onderliggende (multi-)problematiek van de patiënt in een plan voor monitoring, aanvullende diagnostiek en behandeling | | |
| 1. Potentieel risico bij niet goed beheersen van deze EPA | Gevaar voor de kwaliteit van zorg voor de patiënt | | |
| 1. Welke CanMeds competentiegebieden zijn het meest relevant voor deze EPA? | Ø Medisch handelen  Ø Communicatie  Ø Samenwerking  Ο Leiderschap  Ø Kennis en Wetenschap  Ø Maatschappelijk handelen  Ο Professioneel gedrag | | |
| 1. Welke specifieke kennis, vaardigheden en attitudes zijn er nodig om de EPA op het gewenste niveau te beheersen? | 1. Heeft kennis van aandoeningen waarbij de vitale functies potentieel bedreigd kunnen raken, inclusief de achterliggende pathofysiologie 2. Heeft kennis van en vaardigheid met standaard PICU-apparatuur met betrekking tot beademing, bewaking en monitoring van vitale functies en het toepassen hiervan 3. Herkent bedreiging van vitale functies en start behandeling 4. Heeft kennis van de principes van de standaard ic-behandeling 5. Werkt volgens de geldende richtlijnen en protocollen 6. Maakt en (her-)evalueert een behandelplan voor korte en lange termijn 7. Kan diagnostische tests en consulten aanvragen en interpreteren 8. Heeft kennis van de risico’s op complicaties en preventieve maatregelen bij een verblijf op de PICU 9. Stimuleert effectief teamwork op de PICU 10. Onderhoudt een professionele relatie met de patiënt en diens familie 11. Neemt verantwoordelijkheid voor patiëntveiligheid en vraagt op tijd hulp | | |
| 1. Suggesties om voortgang en een ‘entrustment decision’ op te baseren | 1. Korte Praktijk Beoordeling (KPB) 2. Case based discussion (CBD) 3. 360 graden feedback 4. Directly Observed Procedural Skill (DOPS) | | |
| 1. Te behalen supervisie niveau | 4  (jaar 1) | Uitgestelde supervisie | Fellow mag activiteit zelfstandig uitvoeren en rapporteert nadien. Kan geleidelijk overgaan naar ongesuperviseerd uitvoeren van activiteit. |
|  | 5  (jaar 2) | Geeft supervisie | Fellow geeft supervisie aan jonge collegae |
| 1. Expiratiedatum | 2 jaar na het niet meer uitgevoerd hebben van deze EPA | | |

| EPA 3 – Opvang en behandeling van een acuut probleem bij een voorheen stabiele patiënt | | | |
| --- | --- | --- | --- |
| 1. Gedetailleerde beschrijving en beperkingen | **Opvang en behandeling van een acuut probleem bij een voorheen stabiele patiënt** op de PICU  Bijvoorbeeld: accidentele of gefaalde detubatie leidend tot een respiratoire insufficiëntie, hemodynamisch significante bloeding, sepsis met circulatoire instabiliteit, spanningspneumothorax etc.  **De fellow:**   - Herkent, prioriteert en handelt acuut bij een enkelvoudige vitale bedreiging van de patiënt | | |
| 1. Potentieel risico bij niet goed beheersen van deze EPA | Gevaar voor de kwaliteit van zorg voor de patiënt | | |
| 1. Welke CanMeds competentiegebieden zijn het meest relevant voor deze EPA? | Ø Medisch handelen  Ø Communicatie  Ø Samenwerking  Ø Leiderschap  Ο Kennis en Wetenschap  Ο Maatschappelijk handelen  Ø Professioneel gedrag | | |
| 1. Welke specifieke kennis, vaardigheden en attitudes zijn er nodig om de EPA op het gewenste niveau te beheersen? | 1. Heeft kennis van aandoeningen waarbij de vitale functies potentieel bedreigd kunnen raken, inclusief de achterliggende pathofysiologie 2. Heeft kennis van en vaardigheid met standaard PICU-apparatuur met betrekking tot beademing, bewaking en monitoring van vitale functies en het toepassen hiervan 3. Herkent bedreiging van vitale functies en start behandeling 4. Heeft kennis van de principes van de standaard ic-behandeling 5. Werkt volgens de geldende richtlijnen en protocollen 6. Maakt en (her-)evalueert een behandelplan voor korte en lange termijn 7. Kan diagnostische tests en consulten aanvragen en interpreteren 8. Heeft kennis van de risico’s op complicaties en preventieve maatregelen bij een verblijf op de PICU 9. Stimuleert effectief teamwork op de PICU 10. Onderhoudt een professionele relatie met de patiënt en diens familie 11. Neemt verantwoordelijkheid voor patiëntveiligheid en vraagt op tijd hulp | | |
| 1. Suggesties om voortgang en een ‘entrustment decision’ op te baseren | 1. Korte Praktijk Beoordeling (KPB) 2. Case based discussion (CBD) 3. 360 graden feedback 4. Directly Observed Procedural Skill (DOPS) | | |
| 1. Te behalen supervisie niveau | 4  (jaar 1) | Uitgestelde supervisie | Fellow mag activiteit zelfstandig uitvoeren en rapporteert nadien. Kan geleidelijk overgaan naar ongesuperviseerd uitvoeren van activiteit. |
|  | 5  (jaar 2) | Geeft supervisie | Fellow geeft supervisie aan jonge collegae |
| 1. Expiratiedatum | 2 jaar na het niet meer uitgevoerd hebben van deze EPA | | |

| EPA 4 – Opvang en behandeling van een hoog-complexe patiënt met een relatief eenvoudig en behandelbaar acuut probleem | | | |
| --- | --- | --- | --- |
| 1. Gedetailleerde beschrijving en beperkingen | Opvang en/of behandeling van een **hoog-complexe patiënt met een relatief eenvoudig en behandelbaar acuut probleem** op de PICU.  Hiertoe behoren bijvoorbeeld: SMA met een pneumonie, hartfalen bij congenitale/verworven hartafwijking, infectie bij immuun-gecompromitteerde patiënt, etc.  **De fellow:**   - Herkent een acuut probleem en handelt hiernaar, rekening houdend met zowel de oorzaak en potentiele gevolgen van het acute probleem als de impact op de onderliggende aandoening | | |
| 1. Potentieel risico bij niet goed beheersen van deze EPA | Gevaar voor de kwaliteit van zorg voor de patiënt | | |
| 1. Welke CanMeds competentiegebieden zijn het meest relevant voor deze EPA? | Ø Medisch handelen  Ø Communicatie  Ø Samenwerking  Ο Leiderschap  Ø Kennis en Wetenschap  Ο Maatschappelijk handelen  Ø Professioneel gedrag | | |
| 1. Welke specifieke kennis, vaardigheden en attitudes zijn er nodig om de EPA op het gewenste niveau te beheersen? | 1. Heeft kennis van aandoeningen waarbij de vitale functies potentieel bedreigd kunnen raken, inclusief de achterliggende pathofysiologie 2. Heeft kennis van en vaardigheid met standaard PICU-apparatuur met betrekking tot beademing, bewaking en monitoring van vitale functies en het toepassen hiervan 3. Herkent bedreiging van vitale functies en start behandeling 4. Heeft kennis van de principes van de standaard ic-behandeling 5. Werkt volgens de geldende richtlijnen en protocollen 6. Maakt en (her-)evalueert een behandelplan voor korte en lange termijn 7. Kan diagnostische tests en consulten aanvragen en interpreteren 8. Heeft kennis van de risico’s op complicaties en preventieve maatregelen bij een verblijf op de PICU 9. Stimuleert effectief teamwork op de PICU 10. Onderhoudt een professionele relatie met de patiënt en diens familie 11. Neemt verantwoordelijkheid voor patiëntveiligheid en vraagt op tijd hulp | | |
| 1. Suggesties om voortgang en een ‘entrustment decision’ op te baseren | 1. Korte Praktijk Beoordeling (KPB) 2. Case based discussion (CBD) 3. 360 graden feedback 4. Directly Observed Procedural Skill (DOPS) | | |
| 1. Te behalen supervisie niveau | 4  (jaar 2) | Uitgestelde supervisie | Fellow mag activiteit zelfstandig uitvoeren en rapporteert nadien. Kan geleidelijk overgaan naar ongesuperviseerd uitvoeren van activiteit. |
|  | 5  (jaar 3) | Geeft supervisie | Fellow geeft supervisie aan jonge collegae |
| 1. Expiratiedatum | 2 jaar na het niet meer uitgevoerd hebben van deze EPA | | |

| EPA 5 – Opvang en behandeling van een acuut zieke, instabiele, hoog-complexe patiënt | | | | | | |
| --- | --- | --- | --- | --- | --- | --- |
| 1. Gedetailleerde beschrijving en beperkingen | | Opvang en/of behandeling van een **acuut zieke, instabiele hoog-complexe patiënt** op de PICU.  Bijvoorbeeld: refractaire shock, reanimatie, instabiele multi-trauma, etc.  **De fellow:**   - Herkent, prioriteert en stemt de behandeling af van meerdere, simultaan optredende levensbedreigende problemen. Toont hierin leiderschap en stuurt het behandelteam aan - Herkent, prioriteert en stemt de behandeling af van meerdere, simultaan optredende levensbedreigende problemen. Toont hierin leiderschap en stuurt het behandelteam aan | | | | |
| 1. Potentieel risico bij niet goed beheersen van deze EPA | | Gevaar voor de kwaliteit van zorg voor de patiënt | | | | |
| 1. Welke CanMeds competentiegebieden zijn het meest relevant voor deze EPA? | | Ø Medisch handelen  Ø Communicatie  Ø Samenwerking  Ø Leiderschap  Ο Kennis en Wetenschap  Ο Maatschappelijk handelen  Ø Professioneel gedrag | | | | |
| 1. Welke specifieke kennis, vaardigheden en attitudes zijn er nodig om de EPA op het gewenste niveau te beheersen? | | 1. Heeft kennis van aandoeningen waarbij de vitale functies potentieel bedreigd kunnen raken, inclusief de achterliggende pathofysiologie 2. Heeft kennis van en vaardigheid met standaard PICU-apparatuur met betrekking tot beademing, bewaking en monitoring van vitale functies en het toepassen hiervan 3. Herkent bedreiging van vitale functies en start behandeling 4. Heeft kennis van de principes van de standaard ic-behandeling 5. Werkt volgens de geldende richtlijnen en protocollen 6. Maakt en (her-)evalueert een behandelplan voor korte en lange termijn 7. Kan diagnostische tests en consulten aanvragen en interpreteren 8. Heeft kennis van de risico’s op complicaties en preventieve maatregelen bij een verblijf op de PICU 9. Stimuleert effectief teamwork op de PICU 10. Onderhoudt een professionele relatie met de patiënt en diens familie 11. Neemt verantwoordelijkheid voor patiëntveiligheid en vraagt op tijd om hulp | | | | |
| 1. Suggesties om voortgang en een ‘entrustment decision’ op te baseren | | 1. Korte Praktijk Beoordeling (KPB) 2. Case based discussion (CBD) 3. 360 graden feedback 4. Directly Observed Procedural Skill (DOPS) | | | | |
| 1. Te behalen supervisie niveau | | 4  (jaar 2) | Uitgestelde supervisie | | Fellow mag activiteit zelfstandig uitvoeren en rapporteert nadien. Kan geleidelijk overgaan naar ongesuperviseerd uitvoeren van activiteit. | |
| 1. Expiratiedatum | | 2 jaar na het niet meer uitgevoerd hebben van deze EPA | | | | |
| EPA 6 – Opvang, behandeling en/of transport van een acuut zieke patiënt buiten de PICU | | | | | | |
| 1. Gedetailleerde beschrijving en beperkingen | Opvang, behandeling en/of transport van een **acuut zieke patiënt buiten de PICU.**  Hiertoe behoren tenminste: opvang op de SEH, transport (intern en/of extern), etc.  **De fellow:**   - Is zich bewust van en capabel in de opvang en behandeling van een patiënt in een andere omgeving, met een ander team en ander materiaal dan op de PICU. - Herkent, prioriteert en stemt de behandeling af van potentieel levensbedreigende problemen. Toont hierin leiderschap en stuurt het behandelteam aan - Voert adequaat de initiële triage uit, prioriteert, geeft passend advies en consulteert zo nodig tijdig hulp - Heeft kennis van de logistiek en risico’s rondom (inter-) regionale transporten | | | | | |
| 1. Potentieel risico bij niet goed beheersen van deze EPA | Gevaar voor de kwaliteit van zorg voor de patiënt | | | | | |
| 1. Welke CanMeds competentiegebieden zijn het meest relevant voor deze EPA? | Ø Medisch handelen  Ø Communicatie  Ø Samenwerking  Ø Leiderschap  Ο Kennis en Wetenschap  Ο Maatschappelijk handelen  Ø Professioneel gedrag | | | | | |
| 1. Welke specifieke kennis, vaardigheden en attitudes zijn er nodig om de EPA op het gewenste niveau te beheersen? | 1. Heeft kennis van aandoeningen waarbij de vitale functies potentieel bedreigd kunnen raken, inclusief de achterliggende pathofysiologie 2. Heeft kennis van en vaardigheid met apparatuur voor beademing, bewaking en monitoring van vitale functies en het toepassen hiervan zoals die buiten de PICU worden gebruikt 3. Herkent bedreiging van vitale functies en start behandeling 4. Heeft kennis van de principes van de standaard ic-behandeling 5. Werkt volgens de geldende richtlijnen en protocollen 6. Maakt en (her-)evalueert een behandelplan voor korte en lange termijn 7. Kan diagnostische tests en consulten aanvragen en interpreteren 8. Stimuleert effectief teamwork ook buiten de PICU 9. Onderhoudt een professionele relatie met de patiënt en diens familie 10. Neemt verantwoordelijkheid voor patiëntveiligheid en vraagt op tijd hulp | | | | | |
| 1. Suggesties om voortgang en een ‘entrustment decision’ op te baseren | 1. Korte Praktijk Beoordeling (KPB) 2. Case based discussion (CBD) 3. 360 graden feedback 4. Directly Observed Procedural Skill (DOPS) | | | | | |
| 1. Te behalen supervisie niveau | 4  (jaar 2) | | | Uitgestelde supervisie | | Fellow mag activiteit zelfstandig uitvoeren en rapporteert nadien. Kan geleidelijk overgaan naar ongesuperviseerd uitvoeren van activiteit. |
|  | 5  (jaar 3) | | | Geeft supervisie | | Fellow geeft supervisie aan jonge collegae |
| 1. Expiratiedatum | 2 jaar na het niet meer uitgevoerd hebben van deze EPA | | | | | |

| EPA 7 – Communicatie met kind, ouders en/of andere betrokken zorgverleners | | | |
| --- | --- | --- | --- |
| 1. Gedetailleerde beschrijving en beperkingen | Communicatie met kind, ouders en/of andere betrokken zorgverleners  Hiertoe behoren tenminste: shared decision making, mondelinge en schriftelijke overdracht, consulten, brieven, second opinion, verwijzing, MDO, end-of life gesprekken | | |
| 1. Potentieel risico bij niet goed beheersen van deze EPA | Gevaar voor de kwaliteit van zorg voor de patiënt | | |
| 1. Welke CanMeds competentiegebieden zijn het meest relevant voor deze EPA? | Ο Medisch handelen  Ø Communicatie  Ø Samenwerking  Ø Leiderschap  Ο Kennis en Wetenschap  Ø Maatschappelijk handelen  Ø Professioneel gedrag | | |
| 1. Welke specifieke kennis, vaardigheden en attitudes zijn er nodig om de EPA op het gewenste niveau te beheersen? | 1. Is in staat een kind/ouder gesprek te voeren dat aansluit bij de belevingswereld van de gesprekspartner(s) 2. Is in staat een MDO te leiden en de adviezen van consulenten in het behandelplan te integreren zonder hierin de regie te verliezen 3. Draagt zorg voor een behandelplan en neemt hierin het voortouw 4. Neemt verantwoordelijkheid voor patiëntveiligheid en vraagt op tijd om hulp 5. Besteedt aandacht aan een zorgvuldige verslaglegging, overdracht en registratie | | |
| 1. Suggesties om voortgang en een ‘entrustment decision’ op te baseren | 1. KPBs 2. 360 graden feedback 3. DOPS | | |
| 1. Te behalen supervisie niveau | 4  (jaar 1) | Uitgestelde supervisie | Fellow mag activiteit zelfstandig uitvoeren en rapporteert nadien. Kan geleidelijk overgaan naar ongesuperviseerd uitvoeren van activiteit. |
|  | 5  (jaar 2) | Geeft supervisie | Fellow geeft supervisie aan jonge collegae |
| 1. Expiratiedatum | 2 jaar na het niet meer uitgevoerd hebben van deze EPA | | |

| EPA 8 – Uitvoeren van verrichtingen | | | |
| --- | --- | --- | --- |
| 1. Gedetailleerde beschrijving en beperkingen | Uitvoeren van verrichtingen die een PICU-arts dient te beheersen  Hiertoe behoren tenminste: intuberen, (echogeleid) centrale/perifere arteriële/ veneuze lijnen, inbrengen thoraxdrain, etc. | | |
| 1. Potentieel risico bij niet goed beheersen van deze EPA | Gevaar voor de kwaliteit van zorg voor de patiënt | | |
| 1. Welke CanMeds competentiegebieden zijn het meest relevant voor deze EPA? | Ø Medisch handelen  Ø Communicatie  Ø Samenwerking  Ø Leiderschap  Ο Kennis en Wetenschap  Ο Maatschappelijk handelen  Ø Professioneel gedrag | | |
| 1. Welke specifieke kennis, vaardigheden en attitudes zijn er nodig om de EPA op het gewenste niveau te beheersen? | 1. Stelt de indicatie voor een interventie, kent de risico’s en bewaakt het tijdspad 2. Kent de procedures van de interventies/vaardigheden 3. Stemt de interventie af op de onderliggende aandoening, inclusief toepassing van de relevante protocollen en richtlijnen 4. Heeft kennis van preventieve maatregelen en weet eventuele complicaties op te vangen 5. Is zich bewust van de human factors en anticipeert hierop. Past CRM-principes toe in het uitvoeren van de handelingen 6. Neemt verantwoordelijkheid voor patiëntveiligheid en vraagt op tijd om hulp 7. Besteedt aandacht aan een zorgvuldige verslaglegging en registratie | | |
| 1. Suggesties om voortgang en een ‘entrustment decision’ op te baseren | 1. KPBs 2. 360 graden feedback 3. DOPS 4. Eigen verslaglegging interventies/vaardigheden | | |
| 1. Te behalen supervisie niveau | 4  (jaar 2) | Uitgestelde supervisie | Fellow mag activiteit zelfstandig uitvoeren en rapporteert nadien. Kan geleidelijk overgaan naar ongesuperviseerd uitvoeren van activiteit. |
|  | 5  (jaar 3) | Geeft supervisie | Fellow geeft supervisie aan jonge collegae |
| 1. Expiratiedatum | 2 jaar na het niet meer uitgevoerd hebben van deze EPA | | |

| EPA 9 – Managen van complexe situaties (op de PICU) | | | |
| --- | --- | --- | --- |
| 1. Gedetailleerde beschrijving en beperkingen | Managen van complexe, bijzondere situaties (op de PICU)  Hiertoe behoren tenminste: triage en prioritering van het patiënten aanbod voor de PICU, ethische dilemma’s, conflict met ouders, kindermishandeling, stervensbegeleiding, palliatieve zorg, orgaandonatieprocedure, etc. | | |
| 1. Potentieel risico bij niet goed beheersen van deze EPA | Gevaar voor de kwaliteit van zorg voor de patiënt | | |
| 1. Welke CanMeds competentiegebieden zijn het meest relevant voor deze EPA? | Ø Medisch handelen  Ø Communicatie  Ø Samenwerking  Ø Leiderschap  Ø Kennis en Wetenschap  Ø Maatschappelijk handelen  Ø Professioneel gedrag | | |
| 1. Welke specifieke kennis, vaardigheden en attitudes zijn er nodig om de EPA op het gewenste niveau te beheersen? | 1. Stemt proportionaliteit van zorg af op de onderliggende aandoeningen en de medische mogelijkheden 2. Past de relevante protocollen en richtlijnen toe 3. Heeft kennis van palliatieve zorg en de organisatie van een palliatief traject en past dit in de praktijk toe 4. Heeft kennis van orgaandonatierichtlijnen en kan dit traject in goede banen leiden 5. Neemt verantwoordelijkheid voor patiëntveiligheid en vraagt op tijd om hulp 6. Besteedt aandacht aan een zorgvuldige verslaglegging en registratie | | |
| 1. Suggesties om voortgang en een ‘entrustment decision’ op te baseren | 1. KPBs 2. 360 graden feedback | | |
| 1. Te behalen supervisie niveau | 4  (jaar 2) | Uitgestelde supervisie | Fellow mag activiteit zelfstandig uitvoeren en rapporteert nadien. Kan geleidelijk overgaan naar ongesuperviseerd uitvoeren van activiteit. |
|  | 5  (jaar 3) | Geeft supervisie | Fellow geeft supervisie aan jonge collegae |
| 1. Expiratiedatum | 1 jaar na het niet meer uitgevoerd hebben van deze EPA | | |
