# Supplementary material for: Development of entrustable professional activities for paediatric intensive care fellows: A national modified Delphi study
Source: PLoS One. 2021 Mar 18;16(3):e0248565. doi: 10.1371/journal.pone.0248565 (PMC7971696; doi:10.1371/journal.pone.0248565)
Supplement: S2 File — (DOCX) [file pone.0248565.s002.docx]

**DELPHI STUDY**

**PICU EPAs – Round 1**

1. **General demographic questions**

Age: ….in years

Sexe male/ female / prefer not to disclose

Specialty: Paediatrics/ Anaesthesiology

Prior experience on PICU: ….in years

Province: ………………………..

1. **EPA specific questions**

Per individual EPA (n=9) you will be asked to rate whether this EPA reflects an essential core task of a paediatric intensive care physician (indispensability) and whether or not the description of this EPA is sufficiently clear (clarity)

***EPA 1: Assessing and treating a not acutely ill, stable, low complexity patient***

1. *To what extent does this EPA describe an essential task for a PICU physician*

| *none* | *Low* | *Medium* | *High* | *Very high* |
| --- | --- | --- | --- | --- |

1. *The clarity of the description of this EPA is:*

| *Very poor* | *Poor* | *Neither poor nor good* | *Good* | *Very good* |
| --- | --- | --- | --- | --- |

1. *Additional comments for this EPA*

|  |
| --- |

For EPA 2 – 9, identical questions were asked.

1. **Finally**

The current list of EPAs now consists of 9 EPAs:

1. Assessing and treating a not acutely ill, stable, low complexity patient
2. Assessing and treating a not acutely ill, stable, high complexity patient
3. Assessing and treating an acute problem of a previously stable patient
4. Assessing and treating a high complexity patient with a relatively simple and treatable acute problem
5. Assessing and treating an acutely ill, unstable, high complexity patient
6. Assessing and treating an acutely ill patient outside of the PICU
7. Communicating with other healthcare professionals
8. Performing skills essential for PICU physicians
9. Managing complex situations on a PICU
10. *In your opinion, does this list of EPAs reflect all essential professional activities of a PICU physician*

| *Yes* | *No* |
| --- | --- |

1. *If not, which EPAs should be added to the list mentioned above?*

|  |
| --- |

**PICU EPAs – Round 2**

1. **EPA specific questions**

Per revised individual EPA (n=4) you will be asked to rate whether this EPA reflects an essential core task of a paediatric intensive care physician (indispensability) and whether or not the description of this EPA is sufficiently clear (clarity).

***EPA 6:*** Assessing and treating an acutely ill patient outside of the PICU

1. *To what extent does this revised EPA describe an essential task for a PICU physician*

| *none* | *Low* | *Medium* | *High* | *Very high* |
| --- | --- | --- | --- | --- |

1. *The clarity of the description of this revised EPA is:*

| *Very poor* | *Poor* | *Neither poor nor good* | *Good* | *Very good* |
| --- | --- | --- | --- | --- |

1. *Additional comments for this revised EPA*

|  |
| --- |

For EPA 4, 7, 9, identical questions were asked.

**PICU EPAs – Round 3**

1. **EPA specific questions**
2. Do you agree with implementation of this individual EPA?

EPA 1: Assessing and treating a not acutely ill, stable, low complexity patient

| Yes | No |
| --- | --- |

For EPA 2 - 9 the exact same questions were asked

1. Do you agree with implementation of this list of EPAs?
2. Assessing and treating a not acutely ill, stable, low complexity patient
3. Assessing and treating a not acutely ill, stable, high complexity patient
4. Assessing and treating an acute problem of a previously stable patient
5. Assessing and treating a high complexity patient with a relatively simple and treatable acute problem
6. Assessing, treating an acutely ill, unstable, high complexity patient
7. Assessing and treating and/or transporting an acutely ill patient outside of the PICU
8. Communicating with patients, parents/caregivers and/or other healthcare professionals
9. Performing skills essential for PICU physicians
10. Managing complex situations (on a PICU)

| Yes | No |
| --- | --- |
