# Supplementary material for: Development of entrustable professional activities for paediatric intensive care fellows: A national modified Delphi study
Source: PLoS One. 2021 Mar 18;16(3):e0248565. doi: 10.1371/journal.pone.0248565 (PMC7971696; doi:10.1371/journal.pone.0248565)
Supplement: S6 File — (DOCX) [file pone.0248565.s006.docx]

**DELPHI STUDIE**

**PICU EPAs – Ronde 1**

1. **Algemene demografische vragen**

Leeftijd: ….in jaren

Geslacht man/ vrouw / hier wil ik geen antwoord op geven

Moederspecialisme: kindergeneeskunde/ anesthesiologie

Ervaring op PICU: ….in jaren

Provincie: ………………………..

1. **EPA specifieke vragen**

Per EPA (n=9) wordt u nu gevraagd te beoordelen of de betreffende EPA een essentiële kernactiviteit van een kinderintensivist beschrijft en of de betreffende EPA duidelijk omschreven is.

***EPA 1: Opvang en behandeling van een niet-acuut zieke, stabiele, laag-complexe patiënt.***

1. *Deze EPA omschrijft een essentiële kernactiviteit van een kinderintensivist*

| *Volledig mee oneens* | *Mee oneens* | *Neutraal* | *Mee eens* | *Volledig mee eens* |
| --- | --- | --- | --- | --- |

1. *Deze EPA is duidelijk omschreven*

| *Volledig mee oneens* | *Mee oneens* | *Neutraal* | *Mee eens* | *Volledig mee eens* |
| --- | --- | --- | --- | --- |

1. *Ruimte voor opmerkingen over deze EPA*

|  |
| --- |

Over EPA 2 t/m 9 worden exact dezelfde vragen gesteld, alleen de EPAs verschillen.

1. **Tot slot:**

De huidige lijst van PICU EPAs bestaat nu uit:

1. Opvang en behandeling van een niet-acuut zieke, stabiele, laag-complexe patiënt
2. Opvang en behandeling van een niet-acuut zieke, stabiele, hoog-complexe patiënt
3. Opvang en behandeling van een acuut probleem bij een voorheen stabiele patiënt
4. Opvang en behandeling van een hoog-complexe patiënt met een relatief eenvoudig te behandelen acuut probleem
5. Opvang en behandeling van een acuut zieke, instabiele, hoog-complexe patiënt
6. Opvang en behandeling van een acuut zieke patiënt buiten de kinderintensive care
7. Communicatie met andere zorgverleners
8. Uitvoeren van verrichtingen voor een PICU-arts
9. Managen van complexe situaties op de PICU
10. *Geeft deze lijst van EPA’s in uw optiek de essentiële activiteiten van het vak van kinderintensivist volledig weer?*

| *Ja* | *Nee* |
| --- | --- |

1. *Indien u bij de vorige vraag ‘nee’ hebt geantwoord, welke EPA zou er volgens u dan nog moeten worden toegevoegd aan de lijst?*

|  |
| --- |

**PICU EPAs – Ronde 2**

1. **EPA specifieke vragen**

Per gereviseerde EPA (n=4) wordt u nu gevraagd te beoordelen of de betreffende EPA een essentiële kernactiviteit van een kinderintensivist beschrijft en of de betreffende EPA duidelijk omschreven is.

***EPA 4: Opvang en behandeling van een hoog-complexe patiënt met een relatief eenvoudig en behandelbaar acuut probleem***

1. *Deze EPA omschrijft een essentiële kernactiviteit van een kinderintensivist*

| *Volledig mee oneens* | *Mee oneens* | *Neutraal* | *Mee eens* | *Volledig mee eens* |
| --- | --- | --- | --- | --- |

1. *Deze EPA is duidelijk omschreven*

| *Volledig mee oneens* | *Mee oneens* | *Neutraal* | *Mee eens* | *Volledig mee eens* |
| --- | --- | --- | --- | --- |

1. *Ruimte voor opmerkingen over deze EPA*

|  |
| --- |

Over de overige 3 gereviseerde EPAs (6,7 en 9) worden exact dezelfde vragen gesteld, alleen de gereviseerde EPAs verschillen.

**PICU EPAs – Ronde 3**

1. **EPA specifieke vragen**
2. Bent u akkoord met het implementeren van de individuele EPA:

EPA 1: Opvang en behandeling van een niet-acuut zieke, stabiele, laag-complexe patiënt

| Ja | Nee |
| --- | --- |

Voor EPA 2 t/m 9 worden exact dezelfde vragen gesteld.

1. Bent u akkoord met het implementeren van de gehele lijst van 9 EPAs die de kernactiviteiten van een kinderintensivist omschrijven zijn:
2. Opvang en behandeling van een niet-acuut zieke, stabiele, laag-complexe patiënt
3. Opvang en behandeling van een niet-acuut zieke, stabiele, hoog-complexe patiënt
4. Opvang en behandeling van een acuut probleem bij een voorheen stabiele patiënt
5. Opvang en behandeling van een hoog-complexe patiënt met een relatief eenvoudig en behandelbaar acuut probleem
6. Opvang en behandeling van een acuut zieke, instabiele, hoog-complexe patiënt
7. Opvang, behandeling en/of transport van een acuut zieke patiënt buiten de PICU
8. Communicatie met kind, ouders en/of andere betrokken zorgverleners
9. Uitvoeren van verrichtingen voor een PICU-arts
10. Managen van complexe situaties (op de PICU)

| Ja | Nee |
| --- | --- |
